# Recombinant lentogenic Newcastle disease virus expressing Ebola virus GP infects cells independently of exogenous trypsin and uses macropinocytosis as the major pathway for cell entry

**DOI:** 10.1186/1743-422X-10-331

**Published:** 2013-11-09

**Authors:** Zhiyuan Wen, Bolin Zhao, Kun Song, Xule Hu, Weiye Chen, Dongni Kong, Jinying Ge, Zhigao Bu

**Affiliations:** 1 State Key Laboratory of Veterinary Biotechnology, Harbin Veterinary Research Institute, Chinese Academy of Agricultural Sciences, 427 Maduan Street, Harbin 150001, People’s Republic of China

**Keywords:** Recombinant Newcastle disease virus, Ebola virus, Glycoprotein, Virus entry, Macropinocytosis

## Abstract

**Background:**

Using reverse genetics, we generated a recombinant low-pathogenic LaSota strain Newcastle disease virus (NDV) expressing the glycoprotein (GP) of Ebola virus (EBOV), designated rLa-EBOVGP, and evaluated its biological characteristic in vivo and in vitro.

**Results:**

The introduction and expression of the EBOV *GP* gene did not increase the virulence of the NDV vector in poultry or mice. EBOV GP was incorporated into the particle of the vector virus and the recombinant virus rLa-EBOVGP infected cells and spread within them independently of exogenous trypsin. rLa-EBOVGP is more resistant to NDV antiserum than the vector NDV and is moderately sensitive to EBOV GP antiserum. More importantly, infection with rLa-EBOVGP was markedly inhibited by IPA3, indicating that rLa-EBOVGP uses macropinocytosis as the major internalization pathway for cell entry.

**Conclusions:**

The results demonstrate that EBOV GP in recombinant NDV particles functions independently to mediate the viral infection of the host cells and alters the cell-entry pathway.

## Background

Ebola virus (EBOV) causes severe hemorrhagic fever in humans, with a case fatality rate of up to 90% [1,2]. Its high fatality rate and human-to-human spread renders the virus a potential bioterrorism weapon. Currently, there are no licensed vaccines or therapeutic regimens for the disease. A safe and efficient vaccine for EBOV is yet to be developed. EBOV is an enveloped single-stranded negative-sense RNA virus belonging to the family *Filoviridae*[3]. The envelope glycoprotein (GP) of EBOV is an important virulence factor and mediates cell receptor binding and virus–cell membrane fusion [2,4-10]. GP protein also plays a central role in inducing protective neutralizing antibodies in the host [11,12]. Several recombinant GP-expressing viruses have been developed, including replication-defective adenovirus-5 (rAd5) [13,14], replication-competent vesicular stomatitis virus (VSV) [15,16], Newcastle diseases virus (NDV)[17], rabies virus (RV) [18] and human parainfluenza virus type 3 (HPAIV) (Bukreyev et al., 2007).

Newcastle disease virus (NDV) is a member of the genus *Avulavirus* of the family *Paramyxoviridae*. NDV strains are classified as nonvirulent (lentogenic), moderately virulent (mesogenic), or highly virulent (velogenic) in poultry [19]. NDV has two envelope glycoproteins, hemagglutinin-neuramidinase (HN) and fusion protein (F). HN functions sialic acid receptor binding and F induces fusion during cell entry of NDV [20]. Viral virulence is mainly determined by the amino acid sequence at the protease cleavage site of theF precursor [20]. Lentogenic strains contain fewer basic amino acids at this site and can only be cleaved by trypsin-like extracellular proteases, which are largely confined to the respiratory tract, whereas highly virulent strains are cleaved by ubiquitous intracellular proteases, potentially resulting in systemic infections [21]. The attractions of NDV as a vaccine vector for emerging human infectious diseases include: preexisting immunity and maternal antibodies to mammalian paramyxoviruses do not interfere with the infection or replication of NDV because NDV is antigenically distinct from the mammalian paramyxoviruses [22,23]; lentogenic NDV usually shows limited replication in mammalian host cells because it requires a trypsin-like proteinase for the cleavage of the F glycoprotein [20,21,24,25]. Currently, lentogenic NDV strains, such as the LaSota strain, are used as live attenuated vaccines against NDV in poultry [26] and have been actively developed and evaluated as vaccine vectors for the control of human and animal infectious diseases, including influenza [27,28], severe acute respiratory syndrome [29], human parainfluenza [30], highly pathogenic H5N1 [13,31,32], human immunodeficiency virus [33,34], rabies [35], Nipah disease [36], and Rift Valley fever [37]. The safety and efficacy of NDV has been demonstrated in mice [32,36], dogs [35], pigs [36], cattle [38,39], sheep [37], African green and rhesus monkeys [17,30], and humans [40-43]. Recently, a study by DiNapoli et al. showed that a recombinant NDV expressing EBOV GP was immunogenic and caused no abnormalities or disease symptoms after its inoculation into rhesus monkeys [17]. Their study also showed that EBOV GP was incorporated into the recombinant NDV particles, which raised a serious question. Does EBOV GP function biologically normally in the virus particle during cell entry? If so, this entails biosafety concerns regarding the candidate vaccine vector.

In this study, we generated a recombinant lentogenic NDV, based on the LaSota strain, that expresses the EBOV GP protein. Its safety for poultry and mice in vivo, its infection and spreadability among cells in vitro, its sensitivity to anti-NDV and anti-EBOV neutralizing antibodies, and the internalization pathway of this recombinant virus were characterized.

## Results

### Expression of EBOV GP does not increase the pathogenicity of the NDV vector in poultry or mice

**Figure 1:**
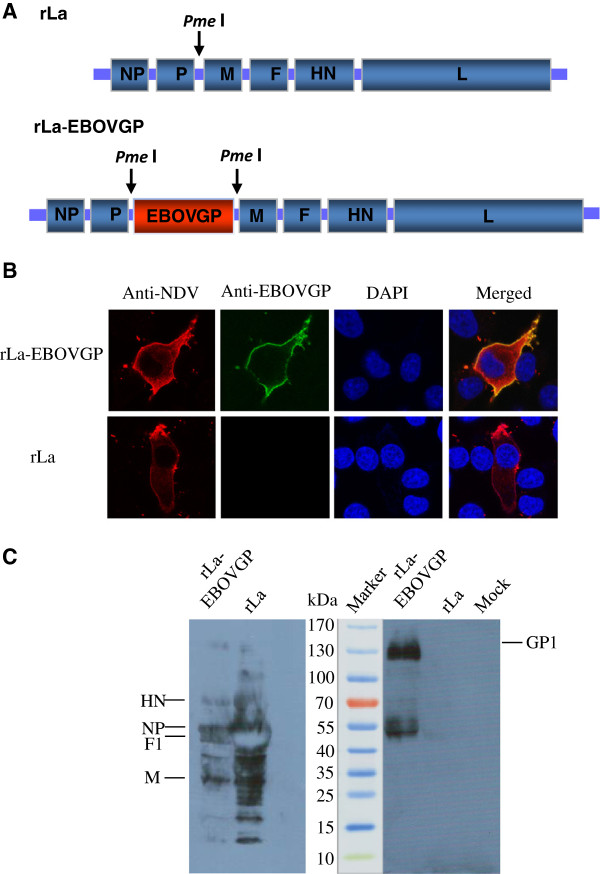
**Generation of recombinant NDV expressing the EBOV *GP *gene. (A)** Schematic representation of the rLa genome, showing the restriction site for endonuclease *Pme*I, introduced between the *P* and *M* genes, and the EBOV *GP* gene inserted into the *Pme*I site. **(B)** Indirect immunofluorescent staining of rLa-EBOVGP-infected BHK-21 cells with chicken anti-NDV antiserum and mouse anti-EBOV GP antiserum, observed with confocal laser microscopy. **(C)** Western blotting of sucrose-gradient-purified rLa and rLa-EBOVGP, hybridized with chicken anti-NDV antiserum and mouse anti-EBOV GP antiserum.

Recombinant NDV expressing the Zaire EBOV GP (rLa-EBOVGP) was generated by inserting the EBOV *GP* gene between the *P* and *M* genes in the genomic cDNA of the NDV LaSota strain (rLa) (Figure 1A). Expression of the *GP* gene was confirmed by indirect confocal immunofluorescent staining of rLa-EBOVGP-infected BHK-21 cells. rLa-EBOVGP-infected BHK-21 cells were stained with mouse anti-EBOV GP antiserum, whereas rLa-infected BHK-21 cells were not stained with the antiserum (Figure 1B). NDV antigens and EBOV GP protein colocalized on the surfaces of the BHK-21 cells, confirming the surface expression of the EBOV GP protein in the rLa-EBOVGP-infected BHK-21 cells (Figure 1B).

rLa-EBOVGP showed similar growth properties to those of rLa, with a maximum titer of 10^9.6^ × 50% embryo infectious doses (EID_50_) at 72 h after inoculation in specific-pathogen-free (SPF) chicken eggs. rLa-EBOVGP retained its low pathogenicity, as a lentogenic strain, in eggs and chickens [44], with a mean death time (MDT) > 140 h, an intracerebral pathogenicity index (ICPI) of 0, and an intravenous pathogenicity index (IVPI) of 0. During the three-week observation period after the mice were inoculated either intramuscularly (*i.m.*) or intranasally (*i.n.*) with a high dose of rLa-EBOVGP, they showed no signs of sickness or death, and did not differ in weight gain from the mice inoculated with rLa. These results suggest that the expression of EBOV GP does not increase the pathogenicity of the NDV vector in poultry or mice.

### EBOV GP is incorporated into the vector virus particles and the recombinant virus rLa-EBOVGP infects cells and spreads among them independently of exogenous trypsin

Previous studies have reported that the envelope glycoproteins of heterogeneous viruses can be expressed by recombinant NDV and incorporated into the viral vector particles [17,35]. To investigate whether the EBOV GP expressed by the recombinant NDV is incorporated into the viral vector particles, the viral particles of rLa-EBOVGP and rLa were purified by sucrose gradient centrifugation and subjected to immunoblotting analysis with mouse anti-EBOV GP antiserum as the primary antibody. A clear GP1 band of ~130 kDa was apparent in the rLa-ZEBOVGP sample but not in the rLa sample. This indicates that the GP protein was expressed by rLa-EBOVGP and incorporated into the recombinant NDV particles, which is consistent with a previous report [17].

**Figure 2:**
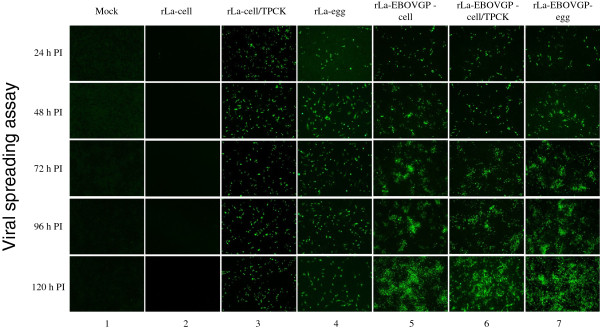
**Viral spreading assay.** Confluent BHK-21 cells were infected with rLa-cell, rLa-cell/TPCK, rLa-EBOVGP-cell, rLa-ZEBOVGP-cell/TPCK, rLa-egg, or rLa-EBOVGP-egg at MOIs of 0.05. The cells were fixed at different time points and detected with chicken anti-NDV antiserum, then incubated with an FITC-conjugated rabbit anti-chicken antibody.

The cleavage of the F glycoprotein is a prerequisite for the infectivity of NDV. Because it is a lentogenic NDV strain, the infectivity of rLa depends on exogenous trypsin-like extracellular proteases [35,36]. To investigate whether the incorporation of EBOV GP alters the infectivity of the recombinant NDV, rLa and rLa-EBOVGP were propagated in eggs (rLa-egg and rLa-EBOVGP-egg, respectively) and in BHK-21 cells with or without TPCK-trypsin (Sigma) in the medium (rLa-cell/TPCK, rLa-EBOVGP-cell/TPCK, rLa-cell, and rLa-EBOVGP-cell respectively). These prepared viruses were then used to infect BHK-21 cells at a multiplicity of infection (MOI) of 0.02–0.05 with no exogenous trypsin in the medium. As expected, rLa-cell did not infect any cells (Figure 2, column 2), whereas rLa-egg and rLa-cell/TPCK infected individual cells but did not spread between the cells. However, rLa-EBOVGP-egg, rLa-EBOVGP-cell, and rLa-EBOVGP-cell/TPCK showed similar infectivity and spreadability in the cells. At 120 h postinfection (PI), more than 90% of cells were infected by all the differently prepared rLa-EBOVGP viruses (Figure 2, columns 3 and 4). These results suggest that the expression and incorporation of EBOV GP allow the vector NDV to infect cells and to spread among the cells independently of exogenous trypsin.

### rLa-EBOVGP is more resistant to NDV antiserum than vector NDV and partially sensitive to EBOV antiserum

**Figure 3:**
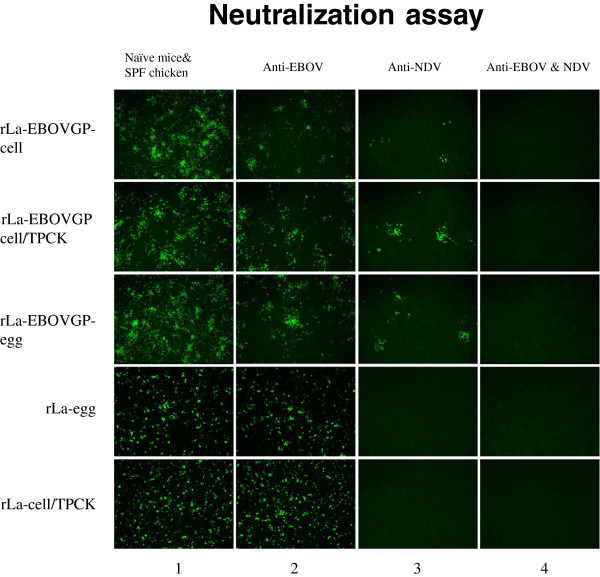
**Neutralization assay.** rLa-cell/TPCK, rLa-EBOVGP-cell, rLa-EBOVGP-cell/TPCK, rLa-egg, and rLa-EBOVGP-egg (each 5 × 10^2^ TCID_50_) were mixed individually with chicken anti-NDV antiserum (diluted 1:100), mouse anti-EBOV GP antiserum (diluted 1:10), or a mixture of chicken and mouse antisera at the same dilutions. The viruses were also mixed with a combination of SPF chicken serum and naïve mouse serum as the mock-treated groups. After incubation, the viruses were allowed to infect BHK-21 cells for 1 h. The cells were fixed 48 h after infection, treated with chicken anti-NDV antiserum, and then incubated with a FITC-conjugated rabbit anti-chicken antibody.

The two envelope glycoproteins of NDV, HN and F, are indispensablefor cell entry, which includes receptor binding and virus-cell membrane fusion. This is the first step in infection and a prerequisite for viral replication [26]. EBOV has only one envelope glycoprotein, which functions in receptor binding and membrane fusion [4-8]. To understand the impact of EBOV GP on the infectivity of the NDV vector, the sensitivities of rLa and rLa-EBOVGP to NDV antiserum and EBOV GP antiserum were evaluated and compared (Figure 3). As expected, both rLa-egg and rLa-cell were resistant to mouse anti-EBOV GP antiserum (diluted 1:10) but were completely neutralized by chicken anti-NDV antiserum (diluted 1:100). However, both anti-NDV antiserum (diluted 1:100) and anti-EBOV GP antiserum (diluted 1:10) only partially neutralized rLa-EBOVGP-cell, rLa-EBOVGP-cell/TPCK, and rLa-EBOVGP-egg (Figure 3, column 2). The anti-NDV antiserum and anti-EBOV GP antiserum reduced the infection with each rLa-EBOVGP virus by about 90% and 60%, respectively (Figure 3, column 3). When the anti-NDV antiserum (diluted 1:100) and anti-EBOV GP antiserum (diluted 1:10) were mixed, infection with rLa-EBOVGP-cell, rLa-EBOVGP-cell/TPCK, or rLa-EBOVGP-cell was completely blocked (Figure 3, column 4). The same dilution of SPF chicken0020serum, naïve mouse serum, or a mixture of SPF chicken serum and naïve mouse serum, used as the controls, showed no neutralization activity against any of the differently prepared rLa or rLa-EBOVGP viruses. These results suggest that the incorporation of EBOV GP into the recombinant viral particle made the NDV vector more resistant to anti-NDV antiserum and more sensitive to anti-EBOV antiserum.

### rLa-EBOVGP uses macropinocytosis as the major internalization pathway for cell entry

**Figure 4:**
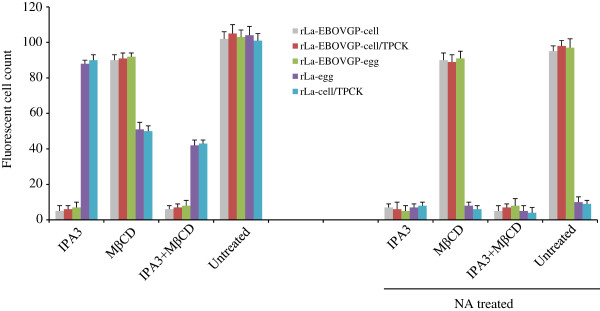
**Chemical inhibition of virus internalization pathways.** BHK-21 cells were pretreated with (right panel) or without (left panel) NA alone or together with inhibitor 10 μM IPA3 or 10 mM MβCD or a mixture of the two chemicals at 37°C for 1 h. The cells were washed and infected with 5 × 10^2^ TCID_50_ of rLa-egg, rLa-EBOVGP-egg, rLa-cell/TPCK rLa-EBOVGP-cell, or rLa-EBOVGP-cell/TPCK at 4°C for 1 h in the presence of the chemical(s). The cells were then washed and incubated at 37°C. At 7 h after infection, the cells were washed, fixed, permeabilized, and immunostained with chicken anti-NDV antiserum, then incubated with a FITC-conjugated rabbit anti-chicken antibody.

NVD envelope glycoproteins HN and F usually bind to receptor and induce virus-cell membrane fusion at neutral pH. A previous study reported that NDV can also partially enter the host cells by caveolae-mediated endocytosis [45], whereas EBOV mainly enters cells via macropinocytosis [46-48] in a GP-dependent manner [46,49]. Because EBOV GP is incorporated into the vector NDV particles and independently mediates the cell entry of rLa-EBOVGP, it is necessary to determine whether the incorporation of the GP protein alters the endocytosis pattern of rLa-EBOVGP during infection. To address this question, we used two chemicals, IPA3 and methyl β-cyclodextrin (MβCD) to treat BHK-21 cells before their infection with the rLa or rLa-EBOV virus. IPA3 inhibits the activation of PAK1 kinase, which is required for macropinocytosis [50,51] and MβCD sequesters cholesterol from the cell membrane, thus inhibiting clathrin- and caveolae-mediated endocytosis [52,53]. As shown in Figure 4, the infection of IPA3-pretreated BHK-21 cells with rLa-EBOVGP-cell, rLa-EBOVGP-cell/TPCK, or rLa-EBOVGP-egg was greatly reduced, whereas IPA3 had no inhibitory effect on the infectivity of rLa-egg or rLa-cell/TPCK. Fewer than 10% of IPA3-pretreated cells were infected with rLa-EBOVGP-cell, rLa-EBOVGP-cell/TPCK, or rLa-EBOVGP-egg compared with the untreated cells. Infection by rLa-EBOVGP-cell, rLa-EBOVGP-cell/TPCK, or rLa-EBOVGP-egg was not inhibited in cells pretreated with MβCD, but their infection by rLa-cell/TPCK and rLa-egg was moderately reduced. Pretreatment with a combination of IPA3 and MβCD almost completely blocked the infection of cells by rLa-EBOVGP-cell, rLa-EBOVGP-cell/TPCK, and rLa-EBOVGP-egg, and also mildly inhibited the infection of cells by rLa-cell/TPCK and rLa-egg. To further testify the role of EBOV GP on the internalization of the recombinant virus, we use 800 mU/ml bacterial neuraminidase (NA, Sigma N2876) to treat cells with chemical inhibitors together with IPA3 or alone before infection. Our preliminary data showed use 800 mU/ml NA to treat cells could block over 90% of NDV infection, thus in this assay we used NA to exclude the influence of HN protein in the internalizaiton of rLa-EBOVGP-egg and rLa-EBOVGP-cell/TPCK. Also shown in Figure 4 (right panel), NA treatment reduced over 90% of rLa-egg and rLa-cell/TPCK infection, while it had almost no reduction on the infection of rLa-EBOVGP-egg, rLa-EBOVGP-cell/TPCK and rLa-EBOVGP-cell. The NA + IPA3 treatment could block 90% of the recombinant viruses from infection, while NA + MβCD had no inhibition on these viruses. These results futher explicated the role of GP on the macropinocytotic internalization of the recombinant viruses. In conclusion, Our results indicate that recombinant rLa-EBOVGP uses macropinocytosis as its major internalization pathway for cell entry, rather than the direct fusion at the cell plasma membrane at neutral pH like NDV.

## Discussion

Using reverse genetics, we generated a recombinant low-pathogenic LaSota NDV that expresses GP of EBOV, designated rLa-EBOVGP, and evaluated its biological characteristics in vivo and in vitro. The introduction and expression of the EBOV *GP* gene did not increase the virulence of the NDV vector in poultry or mice, which is consistent with the results of a previous study in monkeys [17]. EBOV GP was incorporated into the viral particles of rL-EBOVGP and allowed the NDV vector to infect mammalian cells independently of exogenous trypsin.

The restriction of NDV replication in mammalian host cells is one of the most attractive properties of lentogenic NDV in terms of its safety when used as a live vaccine vector in animals and humans, as is also the case for fowlpox virus [54,55] and a modified vaccinia virus Ankara [56,57]. The V protein encoded by the NDV *P* gene functions as an interferon antagonist and is usually less efficient in mammalian cells [58-60]; NDV is usually a strong inducer of the interferon response in mammalian cells and is highly sensitive to the interferon induced in these cells [61,62]. The limited replication in mammalian cells of low-pathogenic NDV, like the LaSota strain, is also determined by its trypsin-dependent infectivity. The trypsin-independent infectivity acquired by rLa-EBOVGP means that this virus behaves like a velogenic NDV in mammalian host cells. Its restricted replication may have to depend on the only defense line in host, the native immunity.

The ability of foreign envelope proteins to function as new cell-entry proteins has also been demonstrated in other enveloped negative-strand RNA viruses [63]. The function of EBOV GP in mediating the cell entry of VSV when the *G* gene had been deleted from the VSV genome could be compensated by several foreign envelope glycoproteins from different viruses in trans or with recombinant expression, including Ebola virus, Marburg virus, Lassa fever virus, Hantaan virus, and Nipah virus [4,6,7,64-67]. Therefore, it is reasonable to infer that the incorporation of EBOV GP into the viral particle may cause the trypsin-independent infectivity of rL-EBOVGP.

So far, very few studies have examined the biological functions of native and foreign envelope glycoproteins when they are incorporated into the same viral particle. Our previous study showed that the G proteins of the rabies virus were incorporated on the surface of a recombinant NDV LaSota particle. An anti-rabies virus antiserum did not reduce the infectivity of the recombinant NDV [35]. The G proteins on the virion surface of the rabies virus did not mediate the infection of cells by the recombinant NDV particle [35]. The reasons for these phenomena are unclear. Another study showed that EBOV GP was incorporated into the viral particle of recombinant human parainfluenza virus 3 (hPIV3) and that the recombinant hPIV3 became more sensitive to neutralizing antibody directed against EBOV than to neutralizing antibody directed against hPIV3 [63]. Because the biological functions of the hPIV3 envelope glycoproteins could not be abolished, it was difficult to clarify whether EBOV GP on the virion surface functioned independently to mediate the infection of cells by the recombinant hPIV3 particle. In the present study, the recombinant NDV rLa-EBOVGP-cell was prepared in BHK-21 cells in the absence of trypsin, so the membrane fusion function of the F protein, essential for viral entry, was completely abolished. Therefore, we can confidently conclude that the EBOV GP that was incorporated into the viral particle independently mediated the cell entry of the recombinant NDV.

As a prototype member of the paramyxoviruses, NDV usually enters host cells by direct fusion at the plasma membrane via a pH-independent mechanism [68-70]. However, NDV can also enter host cells by an endocytic pathway [45]. It had been shown that the cellular entry of EBOV involves a macropinocytosis-like mechanism and subsequent trafficking through early and late endosomes [46,47,71]. Recently, Niemann-Pick C1, a protein involved in the endocytic pathways, has been identified as an important host factor in the cell-entry process [72,73]. In this study, the neutralization assay showed that the mixture of anti-NDV antiserum and anti-EBOV GP antiserum completely blocked the infectivity of rLa-EBOVGP, whereas either anti-NDV antiserum or anti-EBOV GP antiserum only partially blocked the infectivity of this virus. Therefore, we infer that both the NDV envelope proteins F/HN and EBOV GP contribute to the cell entry of rLa-EBOVGP. It is surprising that the inhibition of macropinocytosis almost completely abolished the infectivity of rLa-EBOVGP, whereas it did not markedly reduce the infectivity of rLa. These results indicate that the direct fusion between rLa-EBOVGP and the plasma membrane facilitated by F/HN may not allow viral entry into the cell. EBOV GP plays a major role in the cell-entry process of rLa-EBOVGP, and may predominate over NDV F/HN in the cell-entry function of this recombinant virus. The exact mechanism underlying this predominance requires further investigation. The recombinant virus rLa-EBOVGP provides an interesting model with which to explore the functional interactions between native and foreign envelope glycoproteins in one viral particle.

Although animal tests have shown that recombinant NDV expressing EBOV GP is safe for monkeys [17] and for poultry and mice (this study), safety concerns remain. Because the incorporation of EBOV GP protein into NDV particles significantly alters the behavior of the vector virus, the use of an NDV-vectored EBOV vaccine should be investigated with caution and evaluated rigorously.

## Methods

### Cells and viruses

The BSR-T7/5 cells for virus rescue and the BHK-21 cells for virus growth and titration were maintained in complete Dulbecco’s modified Eagle’s medium (DMEM) containing 10% fetal bovine serum (FBS). The NDV strains were propagated and titrated in 9-day-old SPF embryonated chicken eggs [44] or BHK-21 cells in the presence or absence of TPCK-trypsin (Sigma).

### Plasmid construction and virus rescue

To construct a full-length recombinant genomic cDNA, the cDNA of the *GP* gene of Zaire EBOV was amplified from synthesized cDNA (GenBank accession no. AF086833.2) using the following primers: 5′-GACT**GTTTAAAC**ttagaaaaaaTacgggtagaaC*gccaccATGGGCGTTACAGGAATATTGCAG*-3′ and 5′-CTGA**GTTTAAAC**G*CTAAAAGACAAATTTGCATATACAG*-3′. The *GP* gene was flanked by the restriction site for endonuclease *Pme*I (boldface letters); the NDV gene start sequence (GS, 5′-acgggtagaa-3′) and gene end sequence (GE, 5′-ttagaaaaaa-3′) are included before the optimal Kozak sequence (italic lowercase letters) and the *GP* sequence (italic uppercase letters). The amplified fragment was digested with *Pme*I and then inserted into the P–M intergenic region at nucleotide position 3165 of the NDV genome, as described previously [32]. The resultant plasmid was used for recombinant NDV rescue, as described previously [32]. The expression of EBOV GP was confirmed with an immunofluorescence assay (IFA) and western blotting. The resultant recombinant virus was designated “rLa-EBOVGP”.

### Immunofluorescence assay

NDV infection was detected in cells with IFA with chicken anti-NDV antiserum, as previously described [32]. For the confocal assays, BHK-21 cells were grown in 24-well plates or plated on cover slips in dishes (35 mm diameter) and infected with rLa or rLa-EBOVGP. At 24 h after infection, the cells were fixed in prechilled 3% paraformaldehyde in phosphate-buffered saline (PBS) for 15 min at room temperature, washed three times with PBS, and then blocked with PBS containing 1% (wt/vol) bovine serum albumin at room temperature for 1 h. The cells were then incubated with mouse anti-EBOV GP antiserum (the antiserum was prepared in mice immunized with two doses of recombinant VSV expressing EBOV GP, which was generated in our laboratory) or chicken anti-NDV antiserum for 1 h at room temperature. The cells were then washed three times with PBS containing 0.05% Tween 20 and stained with a fluorescein isothiocyanate (FITC)-conjugated goat anti-mouse antibody (Sigma) or a tetramethylrhodamine isothiocyanate-conjugated rabbit anti-chicken antibody (Sigma) for 30 min. The cells were washed three times with PBS, stained with DAPI, and then analyzed with fluorescence microscopy or confocal laser microscopy. The images were acquired with a Zeiss (Thornwood, NY) Axioskop microscope equipped for epifluorescence with a Sensys charge-coupled device camera (Photometrics, Tucson, AZ) using the IPLab software (Scanalytics, Vienna, VA).

### Western blotting

Egg-propagated rLa and rLa-EBOVGP were purified by sucrose gradient ultracentrifugation. The total protein (5 μg) of each purified virus was subjected to SDS–PAGE under denaturing conditions. After the proteins were transferred from the gel to nitrocellulose membrane, the target band (s) were detected with chicken anti-NDV antiserum or mouse anti-EBOV GP antiserum as the primary antibody and the corresponding horseradish-peroxidase-conjugated goat anti-chicken or goat anti-mouse immunoglobulin G as the secondary antibody. The bands were visualized with ECL Plus Western Blotting Detection Reagents (GE Health Science) on Kodak X-ray film.

### Virus neutralization

Each recombinant virus (5 × 10^2^ TCID_50_) was mixed with chicken anti-NDV antiserum (diluted 1:100) or mouse anti-EBOV GP antiserum (diluted 1:10) or with a mixture of chicken and mouse antisera (at the same dilutions). The viruses were also mixed with a mixture of SPF chicken serum and naïve mouse serum as the mock-treated group. The virus–serum mixtures were incubated at 37°C for 1 h and then used to infect monolayers of BHK-21 cells in a 12-well plate. At 1 h after infection, the supernatants were discarded and the cells were washed three times with DMEM. At 48 h after infection, the cells were fixed, and IFA was performed with chicken anti-NDV antiserum as the primary antibody.

### Assessment of viral pathogenicity

To determine the pathogenicity of rLa-EBOVGP in poultry, MDT, ICPI, and IVPI were determined according to the OIE Manual [44]. To assess the pathogenicity of rLa-EBOVGP in mammalian cells, two groups of 10 six-week-old female Balb/c mice (Vital River, Beijing, China) were inoculated *i.m.* with 10^8^ EID_50_ of rLa-EBOVGP or rLa, and simultaneously *i.n.* with 3 × 10^7^ EID_50_ of rLa-EBOVGP or rLa. The third group of 10 mice was inoculated *i.m.* with 0.1 mL and *i.n.* with 0.03 mL of PBS as the mock-infection control. The mice were monitored daily to detect any weight changes, signs of illness, or death.

### Chemical inhibition of virus internalization pathways

BHK-21 cells were pretreated with either the inhibitor 10 μM IPA3 (Sigma) or 10 mM MβCD (Sigma) alone or together at 37°C. To exclude the influence of HN of vector NDV virus on the internalization of the recombinant viruses, another plate of BHK-21 cells were pretreated with 800 mU/ml bacterial neuraminadase (NA, Sigma N2876) alone or together with the inhibitors at 37°C. At 1 h after treatment, the cells were washed three times and infected with 5 × 10^2^ TCID_50_ of rLa or rLa-EBOVGP at 4°C for 1 h in the presence of the inhibitor(s). The cells were then washed three times on ice. DMEM containing 10% FBS was added and the samples were incubated in 37°C for 7 h. After incubation, the cells were fixed with 3% paraformaldehyde and permeabilized with 0.1% saponin. Cells infected with rLa or rLa-EBOVGP were detected with immunofluorescence assay, as described above. The mean number of flurorecent possitive cells of a minimum of 5 fields of view were counted.The data was expressed as means and standard deviations.

### Ethics statements

The present study was carried out in strict accordance with the recommendations in the Guide for the Care and Use of Laboratory Animals of the Ministry of Science and Technology of the People's Republic of China. The protocol was approved by the Animal Research Ethics Committee of Harbin Veterinary Research Institute, Chinese Academy of Agricultural Sciences (approval numbers 20132085 for chickens and 20132138 for mice).

## Competing interests

The authors declare that they have no competing interests.

## Authors’ contributions

ZB designed and oversaw the experiments. ZB and ZW wrote the manuscript. BZ and JG rescued the recominant viruses and characterized the viruses. ZW carried out the neutralization assay and in vitro cell entry assay. KS, XH, WC and DK carried out the plasmids construction and animal studies. All authors have read and approved the submitted manuscript.

## References

[B1] Feldmann H Geisbert TW Ebola haemorrhagic fever Lancet 2011 10 849 862 10.1016/S0140-6736(10)60667-8 21084112 PMC3406178

[B2] Hoenen T Groseth A Falzarano D Feldmann H Ebola virus: unravelling pathogenesis to combat a deadly disease Trends Mol Med 2006 10 206 215 10.1016/j.molmed.2006.03.006 16616875

[B3] Feldmann H Klenk HD Sanchez A Molecular biology and evolution of filoviruses Arch Virol Suppl 1993 10 81 100 10.1007/978-3-7091-9300-6_8 8219816

[B4] Chan SY Speck RF Ma MC Goldsmith MA Distinct mechanisms of entry by envelope glycoproteins of Marburg and Ebola (Zaire) viruses J Virol 2000 10 4933 4937 10.1128/JVI.74.10.4933-4937.2000 10775638 PMC112022

[B5] Ito H Watanabe S Sanchez A Whitt MA Kawaoka Y Mutational analysis of the putative fusion domain of Ebola virus glycoprotein J Virol 1999 10 8907 8912 10482652 10.1128/jvi.73.10.8907-8912.1999 PMC112919

[B6] Takada A Robison C Goto H Sanchez A Murti KG Whitt MA Kawaoka Y A system for functional analysis of Ebola virus glycoprotein Proc Natl Acad Sci USA 1997 10 14764 14769 10.1073/pnas.94.26.14764 9405687 PMC25111

[B7] Wool-Lewis RJ Bates P Characterization of Ebola virus entry by using pseudotyped viruses: identification of receptor-deficient cell lines J Virol 1998 10 3155 3160 9525641 10.1128/jvi.72.4.3155-3160.1998 PMC109772

[B8] Yang Z Delgado R Xu L Todd RF Nabel EG Sanchez A Nabel GJ Distinct cellular interactions of secreted and transmembrane Ebola virus glycoproteins Science 1998 10 1034 1037 10.1126/science.279.5353.1034 9461435

[B9] Volchkov VE Volchkova VA Muhlberger E Kolesnikova LV Weik M Dolnik O Klenk HD Recovery of infectious Ebola virus from complementary DNA: RNA editing of the GP gene and viral cytotoxicity Science 2001 10 1965 1969 10.1126/science.1057269 11239157

[B10] Yang ZY Duckers HJ Sullivan NJ Sanchez A Nabel EG Nabel GJ Identification of the Ebola virus glycoprotein as the main viral determinant of vascular cell cytotoxicity and injury Nat Med 2000 10 886 889 10.1038/78645 10932225

[B11] Maruyama T Rodriguez LL Jahrling PB Sanchez A Khan AS Nichol ST Peters CJ Parren PW Burton DR Ebola virus can be effectively neutralized by antibody produced in natural human infection J Virol 1999 10 6024 6030 10364354 10.1128/jvi.73.7.6024-6030.1999 PMC112663

[B12] Wilson JA Hevey M Bakken R Guest S Bray M Schmaljohn AL Hart MK Epitopes involved in antibody-mediated protection from Ebola virus Science 2000 10 1664 1666 10.1126/science.287.5458.1664 10698744

[B13] Sullivan NJ Geisbert TW Geisbert JB Shedlock DJ Xu L Lamoreaux L Custers JH Popernack PM Yang ZY Pau MG Immune protection of nonhuman primates against Ebola virus with single low-dose adenovirus vectors encoding modified GPs PLoS Med 2006 10 e177 10.1371/journal.pmed.0030177 16683867 PMC1459482

[B14] Sullivan NJ Hensley L Asiedu C Geisbert TW Stanley D Johnson J Honko A Olinger G Bailey M Geisbert JB CD8+ cellular immunity mediates rAd5 vaccine protection against Ebola virus infection of nonhuman primates Nat Med 2011 10 1128 1131 10.1038/nm.2447 21857654

[B15] Jones SM Feldmann H Stroher U Geisbert JB Fernando L Grolla A Klenk HD Sullivan NJ Volchkov VE Fritz EA Live attenuated recombinant vaccine protects nonhuman primates against Ebola and Marburg viruses Nat Med 2005 10 786 790 10.1038/nm1258 15937495

[B16] Qiu X Fernando L Alimonti JB Melito PL Feldmann F Dick D Stroher U Feldmann H Jones SM Mucosal immunization of cynomolgus macaques with the VSVDeltaG/ZEBOVGP vaccine stimulates strong ebola GP-specific immune responses PLoS One 2009 10 e5547 10.1371/journal.pone.0005547 19440245 PMC2678264

[B17] DiNapoli JM Yang L Samal SK Murphy BR Collins PL Bukreyev A Respiratory tract immunization of non-human primates with a Newcastle disease virus-vectored vaccine candidate against Ebola virus elicits a neutralizing antibody response Vaccine 2010 10 17 25 10.1016/j.vaccine.2010.10.024 21034822 PMC3428043

[B18] Blaney JE Wirblich C Papaneri AB Johnson RF Myers CJ Juelich TL Holbrook MR Freiberg AN Bernbaum JG Jahrling PB Inactivated or live-attenuated bivalent vaccines that confer protection against rabies and Ebola viruses J Virol 2011 10 10605 10616 10.1128/JVI.00558-11 21849459 PMC3187516

[B19] Alexander DJ Newcastle Disease 1989 American Association for Avian Pathologists: Kennett Square, PA

[B20] Peeters BP de Leeuw OS Koch G Gielkens AL Rescue of Newcastle disease virus from cloned cDNA: evidence that cleavability of the fusion protein is a major determinant for virulence J Virol 1999 10 5001 5009 10233962 10.1128/jvi.73.6.5001-5009.1999 PMC112544

[B21] Panda A Huang Z Elankumaran S Rockemann DD Samal SK Role of fusion protein cleavage site in the virulence of Newcastle disease virus Microb Pathog 2004 10 1 10 10.1016/j.micpath.2003.07.003 14643634 PMC7125746

[B22] Charan S Mahajan VM Agarwal LP Newcastle disease virus antibodies in human sera Indian J Med Res 1981 10 303 307 7275226

[B23] Schirrmacher V Haas C Bonifer R Ahlert T Gerhards R Ertel C Human tumor cell modification by virus infection: an efficient and safe way to produce cancer vaccine with pleiotropic immune stimulatory properties when using Newcastle disease virus Gene Ther 1999 10 63 73 10.1038/sj.gt.3300787 10341877

[B24] Morrison T McQuain C Sergel T McGinnes L Reitter J The role of the amino terminus of F1 of the Newcastle disease virus fusion protein in cleavage and fusion Virology 1993 10 997 1000 10.1006/viro.1993.1214 8460504

[B25] Nagai Y Klenk HD Rott R Proteolytic cleavage of the viral glycoproteins and its significance for the virulence of Newcastle disease virus Virology 1976 10 494 508 10.1016/0042-6822(76)90178-1 948870

[B26] Alexander D Newcastle Disease and Other Avian Paramyxoviridae Infections 1997 Ames: Iowa State University Press

[B27] Ge J Tian G Zeng X Jiang Y Chen H Bua Z Generation and evaluation of a Newcastle disease virus-based H9 avian influenza live vaccine Avian Dis 2010 10 294 296 10.1637/8731-032509-ResNote.1 20521648

[B28] Nakaya T Cros J Park MS Nakaya Y Zheng H Sagrera A Villar E Garcia-Sastre A Palese P Recombinant Newcastle disease virus as a vaccine vector J Virol 2001 10 11868 11873 10.1128/JVI.75.23.11868-11873.2001 11689668 PMC114773

[B29] DiNapoli JM Kotelkin A Yang L Elankumaran S Murphy BR Samal SK Collins PL Bukreyev A Newcastle disease virus, a host range-restricted virus, as a vaccine vector for intranasal immunization against emerging pathogens Proc Natl Acad Sci USA 2007 10 9788 9793 10.1073/pnas.0703584104 17535926 PMC1887550

[B30] Bukreyev A Huang Z Yang L Elankumaran S St Claire M Murphy BR Samal SK Collins PL Recombinant newcastle disease virus expressing a foreign viral antigen is attenuated and highly immunogenic in primates J Virol 2005 10 13275 13284 10.1128/JVI.79.21.13275-13284.2005 16227250 PMC1262603

[B31] DiNapoli JM Nayak B Yang L Finneyfrock BW Cook A Andersen H Torres-Velez F Murphy BR Samal SK Collins PL Bukreyev A Newcastle disease virus-vectored vaccines expressing the hemagglutinin or neuraminidase protein of H5N1 highly pathogenic avian influenza virus protect against virus challenge in monkeys J Virol 2010 10 1489 1503 10.1128/JVI.01946-09 19923177 PMC2812327

[B32] Ge J Deng G Wen Z Tian G Wang Y Shi J Wang X Li Y Hu S Jiang Y Newcastle disease virus-based live attenuated vaccine completely protects chickens and mice from lethal challenge of homologous and heterologous H5N1 avian influenza viruses J Virol 2007 10 150 158 10.1128/JVI.01514-06 17050610 PMC1797253

[B33] Khattar SK Samal S Devico AL Collins PL Samal SK Newcastle disease virus expressing human immunodeficiency virus type 1 envelope glycoprotein induces strong mucosal and serum antibody responses in Guinea pigs J Virol 2011 10 10529 10541 10.1128/JVI.05050-11 21849467 PMC3187513

[B34] Carnero E Li W Borderia AV Moltedo B Moran T Garcia-Sastre A Optimization of human immunodeficiency virus gag expression by newcastle disease virus vectors for the induction of potent immune responses J Virol 2009 10 584 597 10.1128/JVI.01443-08 19004953 PMC2612356

[B35] Ge J Wang X Tao L Wen Z Feng N Yang S Xia X Yang C Chen H Bu Z Newcastle disease virus-vectored rabies vaccine is safe, highly immunogenic, and provides long-lasting protection in dogs and cats J Virol 2011 10 8241 8252 10.1128/JVI.00519-11 21632762 PMC3147977

[B36] Kong D Wen Z Su H Ge J Chen W Wang X Wu C Yang C Chen H Bu Z Newcastle disease virus-vectored Nipah encephalitis vaccines induce B and T cell responses in mice and long-lasting neutralizing antibodies in pigs Virology 2012 10 327 335 10.1016/j.virol.2012.06.001 22726244

[B37] Kortekaas J de Boer SM Kant J Vloet RP Antonis AF Moormann RJ Rift Valley fever virus immunity provided by a paramyxovirus vaccine vector Vaccine 2010 10 4394 4401 10.1016/j.vaccine.2010.04.048 20434545

[B38] Kortekaas J Dekker A de Boer SM Weerdmeester K Vloet RP de Wit AA Peeters BP Moormann RJ Intramuscular inoculation of calves with an experimental Newcastle disease virus-based vector vaccine elicits neutralizing antibodies against Rift Valley fever virus Vaccine 2010 10 2271 2276 10.1016/j.vaccine.2010.01.001 20079874

[B39] Khattar SK Collins PL Samal SK Immunization of cattle with recombinant Newcastle disease virus expressing bovine herpesvirus-1 (BHV-1) glycoprotein D induces mucosal and serum antibody responses and provides partial protection against BHV-1 Vaccine 2010 10 3159 3170 10.1016/j.vaccine.2010.02.051 20189484 PMC3428038

[B40] Bukreyev A Collins PL Newcastle disease virus as a vaccine vector for humans Curr Opin Mol Ther 2008 10 46 55 18228181

[B41] Pecora AL Rizvi N Cohen GI Meropol NJ Sterman D Marshall JL Goldberg S Gross P O’Neil JD Groene WS Phase I trial of intravenous administration of PV701, an oncolytic virus, in patients with advanced solid cancers J Clin Oncol 2002 10 2251 2266 10.1200/JCO.2002.08.042 11980996

[B42] Ockert D Schirrmacher V Beck N Stoelben E Ahlert T Flechtenmacher J Hagmuller E Buchcik R Nagel M Saeger HD Newcastle disease virus-infected intact autologous tumor cell vaccine for adjuvant active specific immunotherapy of resected colorectal carcinoma Clin Cancer Res 1996 10 21 28 9816085

[B43] Karcher J Dyckhoff G Beckhove P Reisser C Brysch M Ziouta Y Helmke BH Weidauer H Schirrmacher V Herold-Mende C Antitumor vaccination in patients with head and neck squamous cell carcinomas with autologous virus-modified tumor cells Cancer Res 2004 10 8057 8061 10.1158/0008-5472.CAN-04-1545 15520216

[B44] OIE Manual of Diagnostic Tests and Vaccines for Terrestrial Animals 2011 2011 Paris: Office International des Epizooties

[B45] Cantin C Holguera J Ferreira L Villar E Munoz-Barroso I Newcastle disease virus may enter cells by caveolae-mediated endocytosis J Gen Virol 2007 10 559 569 10.1099/vir.0.82150-0 17251575

[B46] Nanbo A Imai M Watanabe S Noda T Takahashi K Neumann G Halfmann P Kawaoka Y Ebolavirus is internalized into host cells via macropinocytosis in a viral glycoprotein-dependent manner PLoS Pathog 2010 10 e1001121 10.1371/journal.ppat.1001121 20886108 PMC2944813

[B47] Saeed MF Kolokoltsov AA Albrecht T Davey RA Cellular entry of ebola virus involves uptake by a macropinocytosis-like mechanism and subsequent trafficking through early and late endosomes PLoS Pathog 2010 10 e1001110 10.1371/journal.ppat.1001110 20862315 PMC2940741

[B48] Aleksandrowicz P Marzi A Biedenkopf N Beimforde N Becker S Hoenen T Feldmann H Schnittler HJ Ebola virus enters host cells by macropinocytosis and clathrin-mediated endocytosis J Infect Dis 2011 10 Suppl 3 S957 S967 10.1093/infdis/jir326 21987776 PMC3189988

[B49] Swanson JA Shaping cups into phagosomes and macropinosomes Nat Rev Mol Cell Biol 2008 10 639 649 10.1038/nrm2447 18612320 PMC2851551

[B50] Kerr MC Teasdale RD Defining macropinocytosis Traffic 2009 10 364 371 10.1111/j.1600-0854.2009.00878.x 19192253

[B51] Mercer J Helenius A Virus entry by macropinocytosis Nat Cell Biol 2009 10 510 520 10.1038/ncb0509-510 19404330

[B52] Rodal SK Skretting G Garred O Vilhardt F van Deurs B Sandvig K Extraction of cholesterol with methyl-beta-cyclodextrin perturbs formation of clathrin-coated endocytic vesicles Mol Biol Cell 1999 10 961 974 10.1091/mbc.10.4.961 10198050 PMC25220

[B53] Subtil A Gaidarov I Kobylarz K Lampson MA Keen JH McGraw TE Acute cholesterol depletion inhibits clathrin-coated pit budding Proc Natl Acad Sci USA 1999 10 6775 6780 10.1073/pnas.96.12.6775 10359788 PMC21991

[B54] Weingartl HM Berhane Y Caswell JL Loosmore S Audonnet JC Roth JA Czub M Recombinant nipah virus vaccines protect pigs against challenge J Virol 2006 10 7929 7938 10.1128/JVI.00263-06 16873250 PMC1563797

[B55] Odunsi K Matsuzaki J Karbach J Neumann A Mhawech-Fauceglia P Miller A Beck A Morrison CD Ritter G Godoy H Efficacy of vaccination with recombinant vaccinia and fowlpox vectors expressing NY-ESO-1 antigen in ovarian cancer and melanoma patients Proc Natl Acad Sci USA 2012 10 5797 5802 10.1073/pnas.1117208109 22454499 PMC3326498

[B56] Cebere I Dorrell L McShane H Simmons A McCormack S Schmidt C Smith C Brooks M Roberts JE Darwin SC Phase I clinical trial safety of DNA- and modified virus Ankara-vectored human immunodeficiency virus type 1 (HIV-1) vaccines administered alone and in a prime-boost regime to healthy HIV-1-uninfected volunteers Vaccine 2006 10 417 425 10.1016/j.vaccine.2005.08.041 16176847

[B57] Vaccari M Halwani R Patterson LJ Boasso A Beal J Tryniszewska E Hryniewicz A Venzon D Haddad EK El-Far M Antibodies to gp120 and PD-1 expression on virus-specific CD8+ T cells in protection from simian AIDS J Virol 2013 10 3526 3537 10.1128/JVI.02686-12 23325679 PMC3592123

[B58] Park MS Garcia-Sastre A Cros JF Basler CF Palese P Newcastle disease virus V protein is a determinant of host range restriction J Virol 2003 10 9522 9532 10.1128/JVI.77.17.9522-9532.2003 12915566 PMC187425

[B59] Park MS Shaw ML Munoz-Jordan J Cros JF Nakaya T Bouvier N Palese P Garcia-Sastre A Basler CF Newcastle disease virus (NDV)-based assay demonstrates interferon-antagonist activity for the NDV V protein and the Nipah virus V, W, and C proteins J Virol 2003 10 1501 1511 10.1128/JVI.77.2.1501-1511.2003 12502864 PMC140815

[B60] Huang Z Krishnamurthy S Panda A Samal SK Newcastle disease virus V protein is associated with viral pathogenesis and functions as an alpha interferon antagonist J Virol 2003 10 8676 8685 10.1128/JVI.77.16.8676-8685.2003 12885886 PMC167241

[B61] Blach-Olszewska Z Interferon induction by Newcastle disease virus in mice Arch Immunol Ther Exp (Warsz) 1970 10 418 441 5529491

[B62] Brehm G Kirchner H Analysis of the interferons induced in mice in vivo and in macrophages in vitro by Newcastle disease virus and by polyinosinic-polycytidylic acid J Interferon Res 1986 10 21 28 10.1089/jir.1986.6.21 2422299

[B63] Bukreyev A Yang L Zaki SR Shieh WJ Rollin PE Murphy BR Collins PL Sanchez A A single intranasal inoculation with a paramyxovirus-vectored vaccine protects guinea pigs against a lethal-dose Ebola virus challenge J Virol 2006 10 2267 2279 10.1128/JVI.80.5.2267-2279.2006 16474134 PMC1395378

[B64] Kunz S Rojek JM Perez M Spiropoulou CF Oldstone MB Characterization of the interaction of lassa fever virus with its cellular receptor alpha-dystroglycan J Virol 2005 10 5979 5987 10.1128/JVI.79.10.5979-5987.2005 15857984 PMC1091707

[B65] Ogino M Ebihara H Lee BH Araki K Lundkvist A Kawaoka Y Yoshimatsu K Arikawa J Use of vesicular stomatitis virus pseudotypes bearing hantaan or seoul virus envelope proteins in a rapid and safe neutralization test Clin Diagn Lab Immunol 2003 10 154 160 12522053 10.1128/CDLI.10.1.154-160.2003 PMC145270

[B66] Kaku Y Noguchi A Marsh GA McEachern JA Okutani A Hotta K Bazartseren B Fukushi S Broder CC Yamada A A neutralization test for specific detection of Nipah virus antibodies using pseudotyped vesicular stomatitis virus expressing green fluorescent protein J Virol Methods 2009 10 7 13 10.1016/j.jviromet.2009.04.037 19433112 PMC7112920

[B67] Wang X Ge J Hu S Wang Q Wen Z Chen H Bu Z Efficacy of DNA immunization with F and G protein genes of Nipah virus Ann N Y Acad Sci 2006 10 243 245 10.1196/annals.1373.029 17135518

[B68] Lamb RA, Kolakofsky D Fields Virology 2001 3 Philadelphia: Lippincott-Williams & Wilkins

[B69] Hernandez LD Hoffman LR Wolfsberg TG White JM Virus-cell and cell-cell fusion Annu Rev Cell Dev Biol 1996 10 627 661 10.1146/annurev.cellbio.12.1.627 8970739

[B70] Lamb RA Paramyxovirus fusion: a hypothesis for changes Virology 1993 10 1 11 10.1006/viro.1993.1561 8212546

[B71] Quinn K Brindley MA Weller ML Kaludov N Kondratowicz A Hunt CL Sinn PL McCray PB Jr Stein CS Davidson BL Rho GTPases modulate entry of Ebola virus and vesicular stomatitis virus pseudotyped vectors J Virol 2009 10 10176 10186 10.1128/JVI.00422-09 19625394 PMC2747995

[B72] Carette JE Raaben M Wong AC Herbert AS Obernosterer G Mulherkar N Kuehne AI Kranzusch PJ Griffin AM Ruthel G Ebola virus entry requires the cholesterol transporter Niemann-Pick C1 Nature 2011 10 340 343 10.1038/nature10348 21866103 PMC3175325

[B73] Miller EH Obernosterer G Raaben M Herbert AS Deffieu MS Krishnan A Ndungo E Sandesara RG Carette JE Kuehne AI Ebola virus entry requires the host-programmed recognition of an intracellular receptor Embo J 2012 10 1947 1960 10.1038/emboj.2012.53 22395071 PMC3343336

